# Endoscopic retrograde cholangiopancreatography as a diagnostic key tool for biliary atresia: feasibility, safety and accuracy in neonates with cholestatic liver disease

**DOI:** 10.1007/s00431-025-06509-7

**Published:** 2025-12-13

**Authors:** Steffen Hartleif, Michael Esser, Jörg Fuchs, Martin Götz, Florian Graepler, Johannes Hilberath, Toni Illhardt, Hans-Joachim Kirschner, Nisar P. Malek, Andreas Manger, Jürgen Schäfer, Ulrike Schempf, Stephan Singer, Christoph Slavetinsky, Dietmar Stüker, Ilias Tsiflikas, Dörte Wichmann, Ekkehard Sturm, Christoph R. Werner

**Affiliations:** 1 Pediatric Gastroenterology and Hepatology, University Children’s Hospital Tübingen, Hoppe-Seyler-Straße 1, 72076 Tübingen, Germany; 2 Department of Pediatric Surgery and Pediatric Urology, University Children’s Hospital Tübingen, Tübingen, Germany; 3 Department of Gastroenterology, Gastrointestinal Oncology, Hepatology, Infectious Diseases and Geriatrics, University Hospital Tübingen, Tübingen, Germany; 4 Department of Gastroenterology and Oncology, Kliniken Böblingen, Böblingen, Germany; 5 Anesthesiology and Intensive Care Medicine, University Hospital Tübingen, Tübingen, Germany; 6 Department of Diagnostic and Interventional Radiology, Section of Pediatric Radiology, University Hospital of Tübingen, Tübingen, Germany; 7 Institute of Pathology, Department for General and Molecular Pathology, University Hospital Tübingen, Tübingen, Germany; 8 Department of General, Visceral and Transplantation Surgery, University Hospital of Tübingen, Tübingen, Germany

**Keywords:** Neonatal cholestasis, Kasai portoenterostomy, Endoscopic retrograde cholangiopancreatography, Diagnostic algorithm

## Abstract

Biliary atresia (BA) is a rare condition in newborns characterised by inflammation and obliteration of bile ducts, leading to cirrhosis. Early diagnosis and treatment through Kasai portoenterostomy are crucial for native liver survival. While diagnostic methods include clinical assessment, laboratory tests and imaging, no non-invasive test can definitively rule out BA. Therefore, laparotomy with cholangiography remains the gold standard for diagnosis. This study assesses the feasibility, safety and accuracy of endoscopic retrograde cholangiopancreatography (ERCP) for diagnosing BA in newborns. The single-center study was conducted at the University Hospital Tübingen. The study included infants with neonatal cholestasis and suspected BA scheduled for ERCP from 2011 to 2023. We identified 60 jaundiced infants with suspected BA scheduled for diagnostic ERCP with a median age of 50 days. Endoscopy was technically feasible in 58/60 children. In 23 of 58 cases, the bile ducts could not be visualized, suggesting BA. Normal bile duct anatomy was visualized in 35/58 patients, and BA was ruled out. The sensitivity was 100%, the specificity was 92.1%, the negative predictive value was 1.0, and the positive predictive value was 0.87. We did not observe any significant complications post-ERCP (in particular, no bleeding, perforation or pancreatitis). However, in one case (1/60; 2%), protracted weaning after general anesthesia occurred.

*Conclusions*: ERCP is a safe, effective and reliable tool for excluding BA in jaundiced infants. ERCP’s integration into the diagnostic algorithm can reduce the burden of surgical diagnostic procedures and enable early diagnosis and portoenterostomy, thereby improving transplant-free survival.

**Table:** 

**What is known – What is new:** • *Biliary atresia (BA) is the leading cause of obstructive jaundice in neonates, requiring early surgical intervention for optimal outcomes.* • *Current diagnostic approaches are multimodal but lack a definitive, non-invasive method to exclude BA.* • *This study demonstrates that ERCP is not only technically feasible in infants as small as 2.6 kg but also highly reliable in excluding BA with 100% sensitivity, 92.1% specificity and a negative predictive value of 1.0.* • *ERCP is a key diagnostic tool for early exclusion of BA, offering greater diagnostic precision than ultrasound, liver biopsy, or laboratory parameters. *

**Graphical Abstract:**

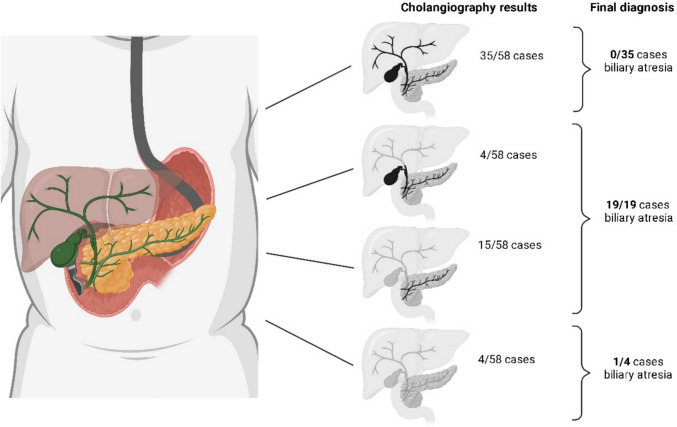

## Introduction

Biliary atresia (BA) is a rare, progressive condition characterised by the obliteration of the biliary ductal system of unclear etiology. It represents a diagnostic challenge in pediatric hepatology [1], in particular since physiological jaundice is common in newborns [2] and timely surgical intervention is critical for cases of BA. BA manifests exclusively in neonates and typically results in the obstruction of bile flow from the liver into the intestine, which, if left untreated, progresses to secondary biliary cirrhosis and liver failure. Since the incidence in Europe [3] and the USA [4] was previously reported as 6–6.5 per 100,000 live births, BA is the most frequently identifiable cause of cholangiopathy leading to jaundice in neonates in the Western world [5]. The diagnostic approach to BA requires a multimodal strategy, given its rarity and clinical presentation overlap with other cholestatic liver diseases [6]. Initial clinical evaluation focuses on identifying cholestatic jaundice that persists beyond the first 2 weeks of life, combined with dark urine and pale stools. Laboratory tests are fundamental in confirming cholestasis by showing elevated direct bilirubin and liver enzyme levels. Further detailed diagnostic exploration is necessary to differentiate BA from other conditions that cause neonatal cholestasis [6], including cystic fibrosis, alpha1-antitrypsin deficiency, Alagille syndrome or progressive familial intrahepatic cholestasis.

Accurate and early diagnosis of BA is critical because prompt restoration of bile drainage by portoenterostomy can help to prevent biliary cirrhosis necessitating liver transplantation or leading to death. Clinical studies emphasize that Kasai portoenterostomy should be performed at the latest by the age of 6 weeks to ensure high native liver survival [7, 8], as the age of the infant and the status of liver fibrosis correlate negatively with native liver survival after Kasai portoenterostomy.

Therefore, accurate diagnostic tools are crucial for excluding or confirming the clinical suspicion of BA. However, current diagnostic tools available for diagnosing BA are either lacking in sensitivity or specificity or require invasive surgical procedures. Previous studies already revealed that endoscopic retrograde cholangiopancreatography (ERCP) is feasible in infants [9–11]. This single-center clinical study aims to evaluate the accuracy of ERCP for the early exclusion of BA and to assess its feasibility and safety in newborn patients. The study also explores how ERCP can contribute to an optimal diagnostic approach and proposes a modified diagnostic algorithm to evaluate infants with neonatal cholestasis employing ERCP at an early stage.

## Methods

We conducted a single-center study on ERCP in cases of neonatal cholestasis at the University Hospital Tübingen from 2011 to 2023. All consecutive infants diagnosed with neonatal cholestasis and suspected BA who were scheduled for ERCP were included in the study.

**Fig. 1:**
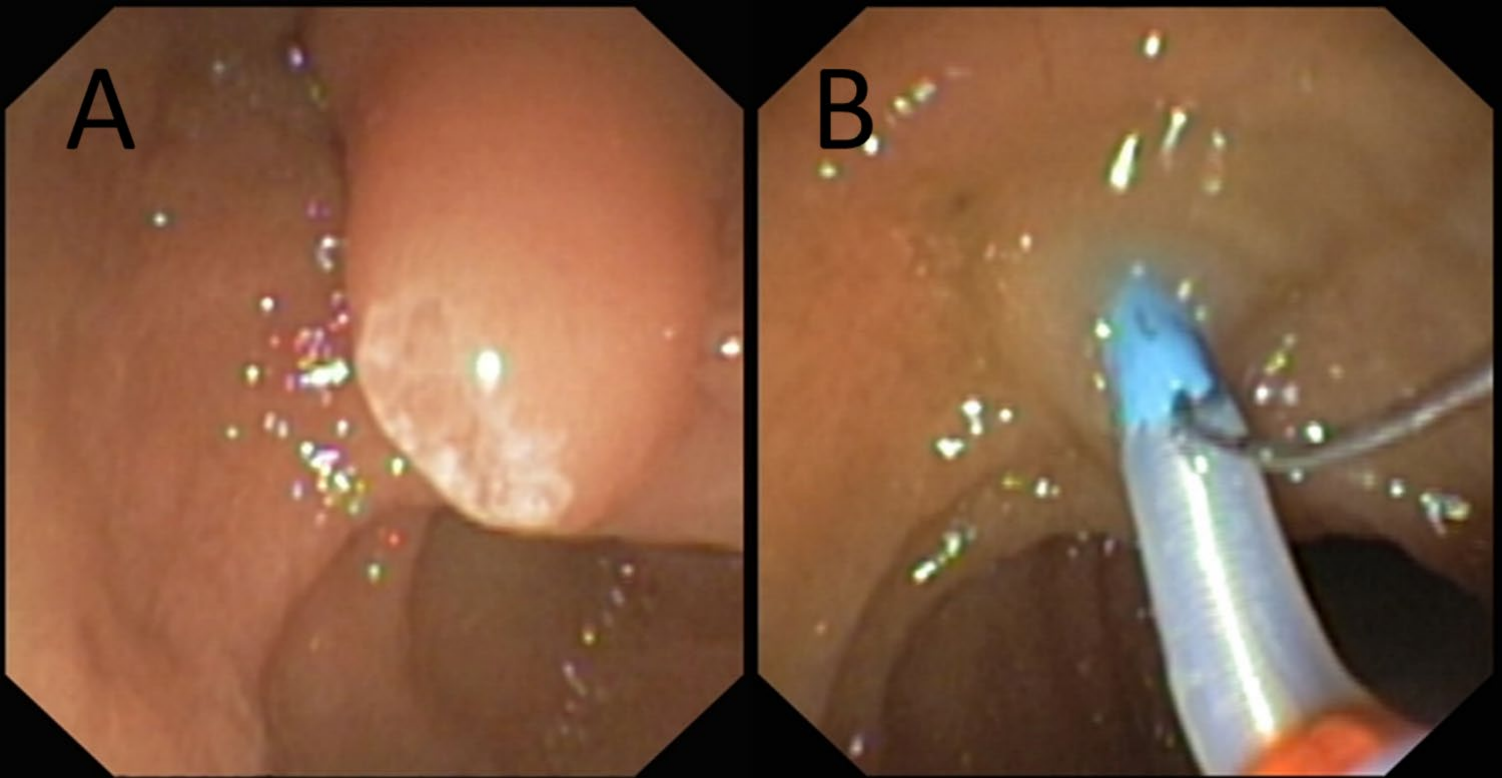
Endoscopic view of the major duodenal papilla before (**A**) and during intubation (**B**)

In all cases, an esophago-gastro-duodenoscopy using a small-bore endoscope (Pentax EG 2490 K, Hoya Corp., Tokyo, Japan; distal end diameter 8 mm) was performed initially to rule out preexisting anatomical abnormalities, such as pyloric stenosis. Then, we performed a diagnostic ERCP using a dedicated pediatric duodenoscope (Olympus PJF 160, Olympus Corp., Tokyo, Japan; distal end diameter 7.5 mm) [12]. For neonatal cholestasis, diagnostic ERCP was performed without sphincterotomy (Fig. 1). The endoscopies were performed by adult gastroenterologists with experience in interventional ERCP in adults and children. The endoscopies were supported by a specialist nurse trained in endoscopy and ERCP, as well as a specialized pediatric anesthesia team. A pediatric hepatologist is present during the ERCP. Results were interpreted in an interdisciplinary approach. The endoscopies were performed in the prone position and under general anesthesia with endotracheal intubation. The anesthesia was balanced with intravenous propofol and remifentanil as well as inhaled sevoflurane. The continuous monitoring of non-invasive vital parameters and end-expiratory CO_2_ is mandatory. We correlated the results of ERCP with patient demographics, clinical presentations, ultrasound results, laboratory findings, liver biopsy results (if available) and clinical outcomes. Data were collected through a review of the patients’ medical charts.

The study was conducted in accordance with the Declaration of Helsinki. All research was approved by the Ethical Committee of the University of Tübingen (project number 819/2023BO2). Informed Consent was not obtained in accordance with the ethical regulations.

### Analysis

Nominal variables were analyzed using Fisher’s exact or chi-square tests for factors with two or more categories. Continuous variables were reported as medians along with either the full range or interquartile ranges (IQRs), as appropriate. The Mann-Whitney *U* test was used for comparison of continuous variables, with a *P* value < 0.05 considered statistically significant. Analyses were performed using IBM SPSS Statistics 28 (IBM Corp., Armonk, New York, USA).

## Results

### Demographics

We identified 60 infants with jaundice, predominantly male (*n* = 41/60; 68%) with a median age of 50 days (ranging from 11 to 105 days), who had undergone ERCP (Table 1) for clinical suspicion of BA. The neonates were selected for ERCP if the diagnostic work-up so far was inconclusive regarding BA. The minimum body weight at the time of the ERCP was 2.6 kg, and the maximum weight was 7.2 kg. All infants presented with pale or acholic stools and direct hyperbilirubinemia, with a median direct bilirubin level of 94.9 µmol/L (range 31–287 µmol/L; normal < 17 µmol/L).

**Table 1:** Patient demographics: vontinuous variables are reported as median [maximal range]

Variable	Statistics; *N* = 60
Median age [days]	50 [11–105]
Median body weight [kg]	4.1 [2.6–7.2]
Male (%)	41 (68%)
Ultrasound suspicious for BA	36 (60%)
Median total bilirubin [µmol]	132.5 [39–416]
Median direct bilirubin [µmol]	94.9 [31–287]
Median gamma-glutamyl transferase [IU/l]	199.5 [30–1757]

### Feasibility and Efficacy of ERCP in neonates with suspected BA

**Fig. 2:**
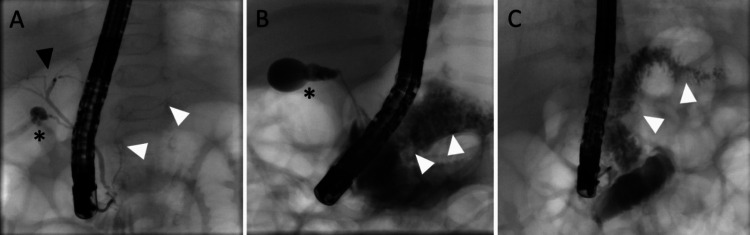
**A** Normal cholangiogram including the common bile duct, left and right hepatic branch (black arrowhead), cystic duct, gallbladder (asterisk) and the subtly contrasted main pancreatic duct (white arrowheads). **B** Patent cystic duct and gallbladder (asterisk), missing common bile duct, heavily contrasted pancreatic parenchyma (white arrowheads) and backwash of contrast agent into the duodenum due to prolonged injection of contrast agent, altogether suggestive of biliary atresia. **C** Absence of the whole biliary system and a contrast-enhanced pancreatic parenchyma (white arrowheads). In (**B**) and (**C**), laparotomy confirmed biliary atresia, and both underwent Kasai’s portoenterostomy

In two children, the papilla could not be reached because a passage into the duodenum could not be achieved due to a narrowed pyloric channel. Thus, ERCP was technically feasible in 58 out of 60 patients (97%): Of those, in 19 out of 58 cases (33%), the biliary duct was either not visualized (*n* = 15/58; 26%) or only partially visualized (*n* = 4/58; 7%), while the pancreatic duct appeared normal, suggesting a diagnosis of biliary atresia (BA). Normal bile ducts were observed in 35 of the 58 cholangiographies (60%), thereby ruling out BA (Fig. 2 and Table 2). In 4/58 patients (7%), neither the biliary duct nor the pancreatic duct could be visualized. Of those four patients, one patient was later diagnosed with BA through laparotomy, whereas in three patients, laparotomy ruled out BA. Cholangiography in three patients revealed hypoplastic intrahepatic bile ducts: One patient was diagnosed with Alagille syndrome, while another had trisomy 21.

**Table 2:** Results of ERCP. A total of 60 patients were included in the study

Patients with neonatal cholestasis		**Outcome**
ERCP technically feasible (major papilla reached)	58/60; 97%	
Pathological Cholangiogram	23/58 (33%)	BA in 20/23 87%
Thereof: no visualization of the biliary duct, cystic duct, and gall bladder; normal visualization of the pancreatic duct	15/58; 26%	BA in 15/15 100%
Thereof: partial visualization of proximal bile ducts, cystic duct, and gall bladder; normal visualization of the pancreatic duct	4/58; 7%	BA in 4/4; 100%
Thereof: no visualization of biliary or pancreatic duct structures	4/58; 7%	BA in 1/4; 25%
Normal cholangiogram	35/58; 60%	BA in 0/35; 0%

The diagnosis of BA was confirmed in 21 cases by laparotomy, inspection and direct cholangiography, and Kasai portoenterostomy was performed.

In summary, ERCP revealed three false-positive patients but no false-negative patients. The sensitivity was 100%, the specificity was 92.1%, the negative predictive value was 1.0 and the positive predictive value was 0.87.

The remaining 39 patients were diagnosed with other conditions after completion of the diagnostic work-up, including whole-exome sequencing: cystic fibrosis (*n* = 3), trisomy 21 (*n* = 2), indeterminate cholestatic hepatopathy (*n* = 2), Alagille syndrome (*n* = 1), alpha-1 antitrypsin deficiency (*n* = 1), PFIC type 2 (*n* = 1), congenital portosystemic shunt (*n* = 1), HHV-6 infection (*n* = 1), CMV infection (*n* = 1), congenital liver fibrosis (*n* = 1), Gaucher disease (*n* = 1) or transient neonatal cholestasis (*n* = 23). Three patients with transient cholestasis had heterozygous variants in genes determining biliary secretion in the hepatocyte; however, they showed no signs of progressive familial intrahepatic cholestasis.

### Safety of ERCP in neonates with suspected BA

Importantly, we did not observe any significant adverse events post-ERCP (in particular, no bleeding, perforation, or pancreatitis). In 10/41 (24%) cases, elevated plasma lipase concentration (> 120U/l = *2xULN*) was detected up to 48 h after ERCP. However, we observed neither clinical signs nor symptoms of pancreatitis, nor did we detect any inflammatory markers, e.g., elevated C-reactive protein. In addition to potential endoscopic complications, we must note that one prolonged weaning after general anesthesia accounted for one out of 60 cases. The neonate presented with respiratory insufficiency and was subsequently admitted to the pediatric intensive care unit. Invasive mechanical ventilation was gradually weaned, resulting in successful extubation within 24 h.

The infants in whom ERCP could not be completed had respective body weights of 2.9 kg and 3.7 kg. The smallest child with a successful ERCP had a body weight of 2.6 kg, without any complications; in this child, a BA was ruled out.

### Ultrasound findings in neonates with suspected BA

Aside from ERCP, other tests were conducted following our local protocol for neonatal cholestasis. In the cohort, all patients had ultrasound examinations to check for abnormalities of the gallbladder shape and wall, as well as the triangular cord sign, which can be associated with BA morphology [13, 14]. In 17 out of 21 confirmed cases, ultrasound predicted BA (sensitivity 81%). However, 17 out of 39 cases without BA showed a pathological ultrasound report (specificity 56%). The triangular cord sign was rarely detected in both groups.

### Laboratory and histological findings in neonates with suspected BA

The total and direct bilirubin levels were significantly higher in children diagnosed with BA (see Table 3 for details). In contrast, levels of gamma-GT and ALT did not differ significantly between the two groups.

**Table 3:** Comparing biliary atresia (BA) versus non-BA diagnosed patients. Histology findings were classified as typical of BA (striking ductular reaction, bile plugs in bile ducts/ductules, portal stromal edema/fibrosis), compatible with BA (mild to moderate degree of ductular reaction, lobular cholestasis with canalicular plugs, mild portal edema/fibrosis) and not compatible with (early) BA (evidence of ductopenia/bile duct paucity, no significant ductular reaction, dominant giant cell transformation, lobular disarray and lobular inflammation). Nominal variables are reported as n (column %), with *P* values from Fisher’s exact tests. Continuous variables are reported as median (interquartile range), with *P* values from Mann-Whitney *U* tests. Bold P values are significant at *P* < 0.05

Final diagnosis	BA (*N* = 21)	Non-BA (*N* = 39)	*p*-value
ERCP			
- Normal cholangiogram	0	35	**< 0.001**
- Pathological cholangiogram	20	3	
ERCP technical not possible	1	1	
Ultrasound gall bladder			
- Normal	**4** (19%)	**22** (56%)	**0.007**
- dysplastic	$$\left.\begin{array}{c}11\\ {}6\end{array}\right\}17\ \left(81\%\right)$$	$$\left.\begin{array}{c}15\\ {}2\end{array}\right\}17\left(441\%\right)$$	
- No gallbladder detected			
Ultrasound with triangular cord sign	4 (19%)	3 (8%)	0.23
ALT [U/l]	95 (65 – 163)	114 (79 – 196)	0.33
Gamma GT [U/l]	270 (121 – 785)	195 (104 – 322)	0.22
Total bilirubin [μmol/l]	178 (144 – 205)	111 (80 – 140)	**< 0.001**
Direct bilirubin [μmol/l]	118 (86 – 150)	80 (62 – 111)	**0.007**
Liver biopsy assessment	*N* = 16	*N* = 27	
- Not compatible with BA	2 (13%)	14 (52%)	**< 0.001**
- Compatible with BA	7 (44%)	13 (48%)	
- Typical of BA	7 (44%)	0	

In 43 children, liver biopsies were performed to identify typical histopathological changes associated with biliary atresia (BA), such as ductular proliferation, bile plugs and liver fibrosis. The liver histology revealed changes typical of BA in seven patients, all of whom were ultimately diagnosed with the condition. Additionally, in 20 patients, the liver histology was compatible with BA; among these, seven received a final diagnosis of BA, and in 13 patients, BA was finally excluded. The sensitivity of the liver biopsy was found to be 88%, while the specificity was 52%. (compare Table 3).

## Discussion

This single-center study aimed to evaluate the feasibility, safety and accuracy of ERCP in the workup of children with neonatal cholestasis and pale stools, with the aim of either excluding or confirming the diagnosis of BA. Even in small infants with a body weight of 2.6 kg, ERCP was successful in excluding or confirming BA with a very high sensitivity and specificity. The negative predictive value of 1.0 underlines the important role of ERCP as a very reliable tool in excluding BA in neonates with unclear neonatal cholestasis. There were three false-positive cases in which the biliary tract was not displayed due to technical challenges in neonates, such as a difficult duodenal angle and compression of the papilla. False pathological findings on ERCP may also result from hypoplastic bile ducts, as observed in Alagille syndrome. Therefore, it is essential to carefully interpret ERCP results and consider supplementary diagnostic approaches, such as direct cholangiography.

Furthermore, ERCP was safe in small neonates with no severe procedure-related complications, including no post-ERCP pancreatitis (PEP), while serum lipase levels were elevated in some cases. Several measures were taken to prevent PEP, including antibiotic prophylaxis, careful intravenous fluid hydration and avoidance of deep intubation of the papilla. The evidence for non-steroidal anti-inflammatory drugs to prevent PEP in infants and children, such as rectal indomethacin [13] or intravenous ibuprofen [14], is not yet clear. Additionally, there was one complication related to anesthesia, emphasizing the importance of a trained pediatric anesthetist in a pediatric ERCP program.

The preoperative liver biopsy results did show a good sensitivity in our cohort. However, the specificity was poor, particularly if the histological changes were mild, but compatible with the possible diagnosis of BA. According to previous international guidelines [5], liver biopsy is a cornerstone in the diagnostic pathway, providing histopathological evidence of bile duct proliferation, portal tract fibrosis and bile plugs, all of which indicate BA. However, the interpretation can be impaired due to the overlapping histological features with other cholestatic liver diseases in infancy [15, 16]. Additionally, changes in liver biopsies can be mild and nonspecific in the early stages of the disease [15, 16]. Thus, our study demonstrates that a liver biopsy alone may be insufficient for for early diagnosis or exclusion of BA.

Therefore, determining the patency of the extrahepatic biliary tree is crucial in neonates with cholestasis and pale or acholic stools to exclude BA at an early stage. As an alternative to ERCP, percutaneous transhepatic cholangiography (PTC) enables visualization of the bile ducts through direct puncture of the bile ducts or gallbladder, followed by the injection of contrast medium [17]. PTC can therefore be an alternative to demonstrate patent bile ducts in infants with neonatal cholestasis if the technique is established. However, only a few small studies on PTC for infants with neonatal cholestasis have been published so far [20, 21], and further studies are needed to evaluate the diagnostic accuracy of this technique. Finally, PTC can only be performed when the gallbladder lumen or the bile duct is accessible [17].

In contrast, hepatobiliary scintigraphy is limited by its low specificity (range, 68.5–72.2%) [18] and the limited etiologic interpretation of negative tracer excretion. Therefore, this technique should be used selectively, and the results of hepatobiliary scanning require cautious interpretation. Similarly, current evidence indicates that magnetic resonance cholangiopancreatography (MRCP) is not effective for diagnosing BA, given that extrahepatic bile ducts may als be absent in nearly 40% of non-cholestatic neonates, resulting in low specificity in the diagnosis [19]. However, the MRCP is helpful for diagnosing other biliary tree disorders in children, such as choledochal cysts [49].

**Fig. 3:**
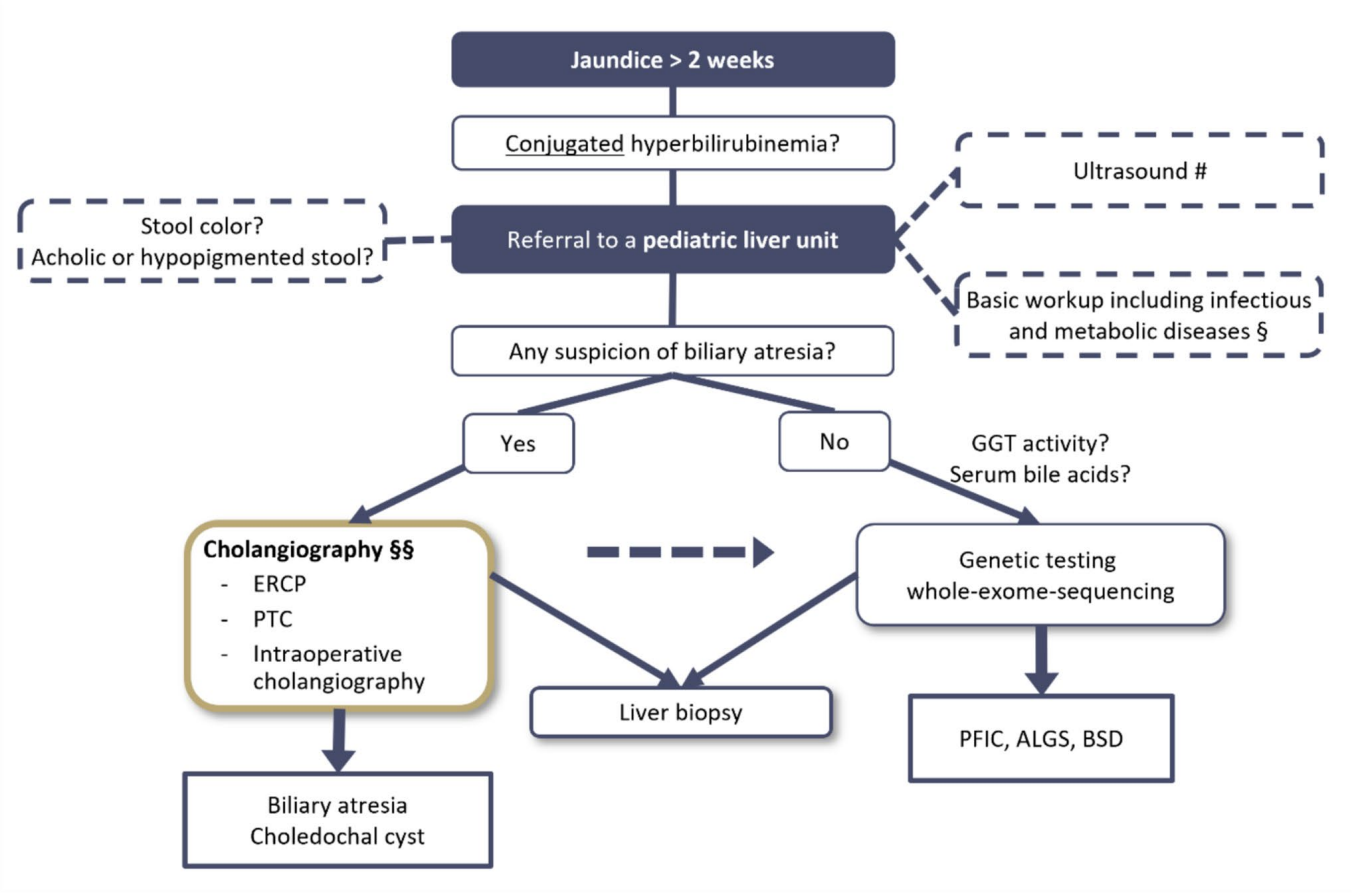
Proposed diagnostic algorithm. # Abdominal Ultrasound with a focus on the morphology of bile ducts and gallbladder, the echogenicity of liver parenchyma, and organomegaly. §Basic assessment: - Exclusion of coagulopathy (check INR and PTT) and exclusion of serious infections, e.g. sepsis, urinary tract infection, viral infection (especially CMV); if suspected toxoplasmosis, rubella or viral hepatitis serology. - Determination of serum bile acids. - TSH, T4, cortisol, alpha-1-antitrypsin levels. - Exclusion of cystic fibrosis (screening result; if necessary, sweat test). - Metabolic diagnostics in plasma and urine: amino acids, organic acids, fatty acids. §§The tool for cholangiography should be chosen based on the degree of suspicion of biliary atresia and local availabity.

A distinct diagnostic pathway (compare Fig. 3) ultimately allows us to exclude biliary atresia (BA) or confirm the need for the Kasai procedure. Early referral to a specialized center with expertise in neonatal cholestasis is strongly recommended. General pediatric centers should refer patients with unclear neonatal cholestasis promptly.

Children with jaundice persisting beyond two weeks and direct hyperbilirubinemia require evaluation in a pediatric liver unit. This evaluation should include laboratory tests—such as prompt coagulation studies—as well as assessments for metabolic and infectious causes, and an abdominal ultrasound. For infants with pale or acholic stools, early imaging of the biliary tree is essential for the timely exclusion or diagnosis of BA. Our goal is to confirm or exclude BA within 7 days of the infant’s presentation at the pediatric liver unit.

Depending on the clinical context, cholangiography can be performed by operative exploration of the biliary tree and direct cholangiography in cases with high suspicion of BA, emphasizing the critical timing of diagnosis. In cases with unclear results, we recommend, based on our data, performing an ERCP, as our results show a high negative predictive value of 100% and a very low complication rate. However, other centers might prefer PTC based on local availability and experience [17]. Once BA has been ruled out, genetic testing using whole-exome sequencing can be valuable in evaluating the genetic causes of neonatal cholestasis [20, 21]. Liver biopsy remains a valuable tool in selected cases. For example, if a lysosomal storage disease is suspected.

Our study demonstrates the high accuracy and safety of ERCP in children with neonatal cholestasis and suspicion of biliary obstruction, highlighting the role of ERCP as the key diagnostic tool for the exclusion of BA in the workup of neonatal cholestasis. However, the following limitations require mentioning in the context of the retrospective nature of the study. The inclusion criteria were neonatal cholestasis and planned ERCP. Patients with the typical clinical picture of BA were directly selected for laparotomy and open cholangiography to confirm the diagnosis of BA. Therefore, these patients could not be included in the study. Further, liver biopsy results were not available in all children, and the impact of additional helpful tests, such as elastography [22] or serum markers, e.g., MMP-7 [23], was not evaluated.

To establish a successful pediatric ERCP program, it is essential to have an experienced specialist in adult or pediatric ERCP, along with the necessary personnel and equipment typically found in pediatric liver centers that perform Kasai procedures or pediatric liver transplants. The team includes a pediatric hepatologist, a pediatric anesthetist, a pediatric intensive care unit and a pediatric hepato-pancreato-biliary surgeon. Periinterventionally, trained pediatric anesthesiologists are mandatory for general anesthesia in these infants.

Designated duodenoscopes are required for performing ERCP in neonates with cholestasis. Unfortunately, the production of duodenoscopes specifically designed for newborns and infants has been discontinued, resulting in limited availability of these essential tools [22], but devices from Olympus are still in circulation and fully serviced. Our findings, along with other successful reports on the role of ERCP in differential diagnostics of BA [9, 23, 24], underscore the need for pediatric and gastroenterology societies to collaborate in improving the accessibility of suitable duodenoscopes for newborns and infants.

In conclusion, this is one of the most extensive single-center studies evaluating ERCP in neonates with suspected BA. It demonstrates that ERCP is not only technically feasible and safe for small infants, but it also possesses high specificity and sensitivity. Within the framework of a targeted diagnostic algorithm, ERCP serves as a reliable tool for excluding BA and, with additional parameters, it may support the early diagnosis of BA.

## Data Availability

The data supporting the findings of this study are not openly available due to reasons of sensitivity and the protection of study participants' privacy. However, anonymised data are available from the corresponding author upon reasonable request. Data are located in controlled-access data storage at the University Hospital Tübingen.

## List of Abbreviations

ALGS: Alagille’s syndrome
BA: Biliary atresia
BASD: Bile acid synthesis disorder
CMV: Cytomegalovirus
ERCP: Endoscopic retrograde cholangiopancreatography
fT4: Free thyroxine
GGT: Gamma-glutamyl transferase
INR: International normalized ratio
MRCP: Magnetic resonance cholangiopancreatography
PEP: Post-ERCP pancreatitis
PFIC: Progressive familial intrahepatic cholestasis
PTC: Percutaneous transhepatic cholangiography
PTT: Partial thromboplastin time
TSH: Thyroid-stimulating hormone
